# Notices of Scarlet Fever in Montgomery County

**Published:** 1832

**Authors:** George W. Branch


### Art. IV.—*Notices of Scarlet Fever in a township of Montgomery County, near Cincinnati, in a letter to Dr. Drake.* By George Branch, M. D.

*Dear Sir,*—I have drawn up and shall send you a few imperfect observations, on the Scarlet Fever, as it appeared in Montgomery and its vicinity. In doing so I am not certain that any thing original will be offered: but, to say the least, it is a portion of the medical history of the west, and perhaps may disprove or confirm opinions already extant.

With regard to the cause of the disease I can say nothing very definite. If its origin was not atmospheric I cannot account for it. Sometimes one or two among half a dozen children, were attacked, while the remainder escaped. It appeared in those who had not been exposed to the disease in others—and those who had been exposed did not often take it.

I observed that most of the attacks occurred immediately after rains, which cleared up warm, with a close and confined atmosphere. The Epidemic was not entirely confined to children, for I saw one adult who had the disease completely developed;—the true scarlet eruption—the anginose affection—and the intense heat of the skin were all present. Many others had the anginose affection only.

Some of the cases were very mild, being attended with a moderate degree of fever—swelling of the parotid glands and tonsils—a thickly coated tongue—no eruption—finishing its course in a week. Others were attended with a scarlet eruption, great heat and itching of the skin, and a very sore throat; the fever generally continuing from ten to fifteen days, and desquamation of the cuticle following the decline of the eruption. Sometimes the efflorescence was not diffused over the body, but came out in irregular patches on different parts. It frequently disappeared and reappeared at uncertain times. In most of the cases the arterial excitement was very strongly developed. During the stage of excitement, head-ache and delirium were common symptoms. In some cases great præcordial oppression was present. From the commencement of the fever a dull pain with stiffness was felt under the ears—under the angles of the jaw, and in the muscles of the neck. At this time, the tonsils, fauces, palate, and uvula were slightly swelled and red. In the course of the disease the voice was hoarse, deglutition painful, and the breathing difficult. When the disease was severe, ulcerations formed in the throat, which were converted into sloughs, that separated on the decline of the fever. In one case the sloughs enlarged—changed to a brown color, and discharged a sanious fluid: the glands of the neck being swelled enormously.

Sequela.—Various troublesome disorders followed the subsidence of the fever. Dropsical effusions were the most common. Indeed I saw but very few cases that recovered without swelling of the extremities. Some in a few weeks from the decline of the fever, would pass into a torpid state, with great disposition to sleep:—no appetite—slight febrile attacks in the afternoon—moderate anasarca—urine dark in color, and depositing a sediment which resembled coal-dust—bowels torpid, requiring large cathartic doses to move them—the skin and tunica albuginea deeply tinged with yellow. These cases were very lingering, but the patients recovered under the use of brisk mercurial purges. In some a discharge of pus from the ear with partial deafness followed. For several weeks, exposure to cold reproduced glandular swellings.

Treatment. Most of the cases required a prompt anti-phlogistic course. Bleeding—antimonial vomits, and afterwards, tartar emetic in broken doses, to curb the action of the heart and arteries—blisters, if much glandular tumefaction existed—gargles of vitriolic acid, and a solution of chloride of lime—cathartics and aperients, and if much heat and itching of the skin, cold effusions, were the remedies which seemed most beneficial.

The dropsies which came on after the decline of the fever, were connected with a phlogistic state of the system. The pulse was quick, sharp and frequent—the skin dry and harsh, and warmer than natural—bowels torpid and urine high-colored and small in quantity. Here purgatives, diuretics—refrigerant diaphoretics, and the warm bath, were used with manifest advantage.

I will relate a case which had some peculiarities. A child, three or four years old, when I first saw it, thirty hours after the attack, had a pulse at the wrist scarcely perceptible—the eruption pale in color—the extremities cold—the upper surface of the tongue, fauces and tonsils of a deep red—the tongue covered with a brown fur, with the red and enlarged papillæ showing through it—the eyes dull, and the respiration oppressed. Stimulants, both external and internal—warm ptizans—calomel in large doses, and stimulating enemata, succeeded in restoring the circulation to the surface and extremities. On the third day, ash-colored ulcers appeared on the tongue and in the fauces; and the glands of the neck were much tumefied, the arterial action and heat of the surface moderate. On the fifth day, the ulcers were converted into black and fetid sloughs. The glandular swellings continued until they reached the orbits of the eyes—the ears—the clavicles, and crossed the trachea; both the breathing and deglutition being rendered difficult. On the seventh day, the swelling began to decline—the cuticle to desquamate—the pulse became more regular and fuller, and every thing seemed for the better until the tenth day, when the little patient manifested a strong disposition to sleep. The following night I was called in haste, and found the breathing laborious and difficult—the face and lips of a dark livid tinge—pulse small, frequent, and intermitting—deglutition very difficult—involuntary stools—extremities cold—tongue covered with a dry and black fur—some of the sloughs in the fauces had separated and left red ulcerated surfaces, which again turned black. Respiration soon became quick and short, succeeded by stupor, in which the little patient expired. In three or four hours after death, putrefaction followed.

You are at liberty to make such use of the foregoing observations as you may deem proper. They are copied from notes which were taken during the progress of the disease, and were only intended to be a history of facts.

Your obliged friend and servant,

GEO. W. BRANCH.

DANIEL DRAKE, M. D.

